# From crash evidence to injury prevention: factors associated with injury severity in heavy vehicle crashes involving vulnerable road users in Pakistan

**DOI:** 10.3389/fpubh.2026.1895759

**Published:** 2026-10-06

**Authors:** Amjad Pervez, Muhammad Junaid, Md. Kamrul Islam, M. M. Hafizur Rahman

**Affiliations:** 1 Institute of Human Factors and Ergonomics, College of Mechatronics and Control Engineering, Shenzhen University, Shenzhen, China; 2 School of Transportation and Logistics, Southwest Jiaotong University (SWJTU), Chengdu, China; 3 Department of Civil and Environmental Engineering, College of Engineering, King Faisal University, Al Ahsa, Saudi Arabia; 4 Department of Computer Networks and Communications, College of Computer Sciences and Information Technology (CCSIT), King Faisal University, Al Ahsa, Saudi Arabia

**Keywords:** heavy vehicles, injury prevention, injury severity, road traffic injuries, unobserved heterogeneity, vulnerable road users

## Abstract

Crashes involving heavy vehicles and vulnerable road users (VRUs) represent a serious public health challenge in low- and middle-income countries, where road traffic injuries contribute substantially to injury-related morbidity and mortality. However, the existing literature remains limited, as most studies focus on single VRU groups and are concentrated in high-income settings. As a result, little is known about injury severity in mixed-traffic environments such as Pakistan, where pedestrians, motorcyclists, and motorized rickshaw users interact with heavy vehicles under substantially different roadway and traffic conditions. To address this gap, this study investigates the factors associated with injury severity in crashes involving heavy vehicles and VRUs in Pakistan using 1,007 anonymized emergency-service crash records collected by Rescue 1122. A random parameters binary probit model with heterogeneity in means and variances was estimated to account for unobserved heterogeneity and variation in the effects of explanatory variables across crashes. The analysis indicates that male VRUs are less likely to sustain severe injuries, whereas VRUs aged 25 years or younger face a significantly higher probability of severe injury. Crashes occurring during weekends, at night, and under rainy conditions are associated with a higher probability of severe injury. Pedestrian and motorcyclist involvement, as well as distraction, are also associated with a substantially higher probability of severe injury in crashes involving heavy vehicles. In addition, primary roads, two- and three-lane roads, vertical curves, sidewalks, and access points are all associated with a higher probability of severe injury. The random parameter estimates further indicate that the effects of nighttime and distraction exhibit significant heterogeneity across crashes, with nighttime having a stronger effect in pedestrian-related crashes, at access points, and under rainy conditions. The results suggest the need for targeted interventions focusing on the protection of pedestrians and motorcyclists, younger road users, high-risk roadway environments, and context-sensitive enforcement and engineering measures on corridors with frequent heavy vehicle movement. These findings provide evidence to support injury prevention strategies, inform public health and transportation policies, and contribute to achieving safer and more equitable mobility systems in Pakistan and other low- and middle-income countries facing similar road safety challenges.

## 1 Introduction

Heavy vehicles play a pivotal role in freight transportation, acting as the backbone that connects suppliers and recipients across regions and countries (1, 2). They provide important operational advantages, ensuring the efficient movement of goods across vast distances and within multimodal supply chains (1, 2). These advantages, together with the rapid expansion of global commerce and increasing freight demand, have been accompanied by a substantial rise in the number of heavy vehicles on roads. While this growth has contributed substantially to economic development, it has also raised concerns regarding road traffic injuries, which remain a major public health issue and a leading cause of death and disability worldwide. The increase has been particularly pronounced over the past two decades. For instance, in the United States, the overall count of trucks rose by 111%, increasing from 85,175,000 in 2002 to 179,818,000 in 2021 (3), while more than 320,000 trucks were manufactured in the United States in 2022, reflecting an approximate 11% increase compared to 2021 (4). Similar trends are evident in low- and middle-income countries, where India’s truck fleet grew from 246,000 in 2021 to 327,000 in 2022, representing a 33% increase, while in Bangladesh the number of registered trucks rose from 4,719 in 2020 to 5,789 in 2021, representing an overall increase of approximately 23% (5, 6). This growth has generated major economic and logistical benefits, but has also created important safety challenges, particularly in urban areas and local communities. Heavy vehicles account for a disproportionate share of road traffic fatalities in several countries. For instance, in the United States, they represent 23 and 21% of all fatalities in North Dakota and Wyoming, respectively, while in Canada, they were involved in approximately 20% of all road traffic fatalities in 2022 (7, 8). The burden is also substantial in low- and middle-income countries, where heavy vehicles account for approximately 40% of road traffic fatalities in India and 34% of fatal crashes on Malaysia’s PLUS highways, which serve as key corridors for long-distance travel and freight transportation (9, 10).

Beyond the overall increase in heavy vehicle-related fatalities, an important concern is their concentration in urban areas and on local roads, which represent high-risk environments from a public health and injury prevention perspective due to dense human exposure and complex mobility interactions. Prior studies (11, 12) have shown that a large proportion of heavy vehicle-related fatalities occur in urban settings and on arterial roads, where the complexity of the transportation environment is associated with greater safety risk. This pattern is partly explained by the size and maneuverability limitations of heavy vehicles, which become more critical in urban environments characterized by congestion, limited road space, and frequent interactions among different road users. These risks are particularly pronounced during last-mile urban delivery operations, where heavy vehicles interact more frequently with vulnerable road users (VRUs) (13). Heavy vehicles, due to their size and weight, are associated with disproportionately severe injury outcomes among VRUs (14). Evidence from high-income countries highlights the magnitude of this problem. In the United States, fatalities involving VRUs in crashes with heavy vehicles increased from 537 in 2012 to 755 in 2022, representing a 40.6% increase (15). Similarly, in Australia, VRUs accounted for 41 (25%) of the 163 fatalities reported in heavy-vehicle crashes in 2021 (16). Beyond their frequency, such interactions are also associated with greater injury severity for VRUs. For instance, prior research (17) found that pedestrians involved in crashes with heavy vehicles were three times more likely to sustain severe injuries than those involved in crashes with passenger vehicles, while existing research (18) reported that pedestrians face a 50% greater risk of fatal injury in crashes involving heavy vehicles than in those involving passenger cars. These findings highlight the disproportionate risks that heavy vehicles pose to VRUs, particularly in complex urban traffic environments, and reinforce the need for evidence-based public health interventions aimed at reducing exposure and mitigating injury severity in high-risk urban settings.

The burden associated with interactions between heavy vehicles and VRUs is particularly severe in low- and middle-income countries, where such crashes constitute a critical public health concern, as road traffic injuries are a leading cause of premature mortality, disability, and health-system burden. This burden is further intensified because rapid motorization has often outpaced the development of safe transport systems, effective injury prevention strategies, and adequately resourced emergency response services. For instance, in India, 28,706 VRUs were killed in crashes involving heavy vehicles in 2023, accounting for 16.6% of the total 172,890 road traffic fatalities reported nationwide (19). A similar pattern is evident in Pakistan, where previous studies have shown that heavy vehicles pose substantial risks to multiple VRU groups. In particular, crashes involving heavy vehicles have been associated with serious and fatal injuries among motorcyclists (20, 21), severe injuries in crashes involving motorized rickshaws (22), and a significantly higher risk of severe injuries among pedestrians involved in collisions with heavy vehicles (23). Collectively, these findings underscore that the injury burden associated with heavy vehicle–VRU interactions is not confined to a single road user group but reflects a broader and systemic public health challenge affecting multiple vulnerable populations. In low- and middle-income countries, and especially in Pakistan, this burden is shaped by a combination of behavioral, infrastructural, and systemic factors. Risky driving behaviors, such as speeding, aggressive overtaking, noncompliance with seatbelt and helmet laws, lane indiscipline, and disregard for traffic signs, have been widely documented as major contributors to road crashes (24, 25), while weak enforcement of traffic regulations remains an additional road safety concern (26). In addition, poor road infrastructure, including a lack of pedestrian facilities, the absence of dedicated lanes for motorcyclists, and conflicts with roadside vendors, is associated with additional road safety risks (27–29). Moreover, limitations in public transportation systems are associated with greater reliance on private and informal transport, particularly motorcycles and motorized rickshaws, which are also associated with substantial road safety risks (22, 28, 30). Similarly, mixed traffic conditions, which are prevalent in low- and middle-income countries, have been associated with more frequent traffic conflicts, reduced predictability of road user behavior, and a greater likelihood of severe injury outcomes (31, 32). Furthermore, the lack of medical facilities and limited access to emergency medical services and trauma care are associated with poorer post-crash outcomes and a higher risk of fatality (33, 34). These health-system constraints represent an important component of the public health burden associated with road traffic injuries in low- and middle-income countries.

Despite the growing public health importance and substantial injury burden associated with interactions between heavy vehicles and VRUs, the available research remains limited. Existing studies have examined heavy vehicle interactions with specific VRU categories, including pedestrians (17, 35), cyclists (14), and motorcyclists (36). In addition, some researchers have considered pedestrians and cyclists together (14, 37–39), while another study jointly examined cyclists and electric-bike users in crashes involving heavy vehicles (35). To the best of the authors’ knowledge, only one study (40) has examined the severity of injuries resulting from interactions between heavy vehicles and multiple major categories of VRUs, namely pedestrians, cyclists, and motorcyclists, in a high-income country. This highlights a critical gap in the international public health and transportation safety literature, where evidence remains fragmented and largely limited to single VRU groups rather than integrated analyses. More importantly, motorized rickshaw users, who constitute an important and distinctive vulnerable group in many low- and middle-income countries, are almost entirely absent from the existing literature. A second gap concerns contextual relevance. Much of the available evidence is derived from high-income-country settings, even though traffic composition, roadway design, enforcement conditions, and road user behavior differ substantially in mixed-traffic environments such as Pakistan. As a result, findings from high-income settings may not be directly generalizable to low- and middle-income countries. This limitation is particularly important from a public health perspective because effective injury prevention strategies require context-specific evidence that reflects local exposure patterns, infrastructure conditions, behavioral factors, and healthcare system capacities. A third gap is that important explanatory factors remain underexplored in the literature on heavy vehicle and VRU crash severity. In particular, seasonal variation and detailed roadway infrastructure characteristics, such as road alignment, lane width, median type, the presence of access points, sidewalks, and U-turns, have received limited attention. This omission is particularly important in Pakistan and similar low- and middle-income countries, where mixed traffic, weak lane discipline, informal roadside activities, and inconsistent enforcement may interact with these factors in ways that shape crash outcomes. These factors are closely related to conflict opportunities, vehicle maneuvering space, visibility conditions, and VRU exposure levels, yet their role in shaping injury severity outcomes in crashes involving heavy vehicles remains poorly understood from a public health and injury prevention standpoint.

In response to these gaps, the present study investigates the factors associated with injury severity in crashes involving heavy vehicles and VRUs in Pakistan, with the aim of generating empirical evidence to inform road traffic injury prevention strategies and strengthen public health policies in low-resource settings. The study addresses the limited international evidence by jointly examining multiple VRU groups within a single analytical framework, while also responding to the shortage of context-specific evidence from mixed-traffic settings in low- and middle-income countries, where infrastructure, enforcement, and healthcare conditions differ substantially and may shape injury outcomes. In particular, the study includes pedestrians, motorcyclists, and motorized rickshaw users, thereby broadening the scope of heavy vehicle and VRU safety analysis in a way that is more representative of Pakistan’s traffic environment. Methodologically, the study applies a random parameters model with heterogeneity in means and variances to analyze emergency-service crash records obtained from Rescue 1122, allowing unobserved heterogeneity and complex variation in the effects of explanatory variables to be captured more effectively. Substantively, the analysis incorporates several factors that have received limited attention in previous heavy vehicle and VRU studies, including road alignment, lane width, median type, the presence of access points, sidewalks, U-turns, and seasonal variation. In this way, Pakistan is treated not only as the empirical context but also as an analytically important case for generating insights relevant to other low- and middle-income countries with similar traffic systems, where reducing the public health burden of road traffic injuries requires context-specific, evidence-based interventions and a stronger integration of transport safety research within public health decision-making frameworks. The study therefore addresses the limited international evidence and the shortage of context-sensitive research from low- and middle-income countries and contributes region-specific evidence on heavy vehicle and VRU crash severity and offers a stronger basis for targeted infrastructure planning, enforcement strategies, and traffic management interventions in Pakistan and other countries with similar traffic and roadway conditions, ultimately supporting global efforts to reduce road traffic injury burden and advance population health outcomes.

## 2 Literature review

### 2.1 Empirical findings on factors associated with heavy vehicle–VRU crash severity

The severity of injuries sustained in heavy vehicle–VRU collisions has been associated with various factors, including demographic factors, temporal and weather conditions, behavioral factors, the types of vehicles involved, and roadway geometric characteristics, as summarized in Table 1. The table indicates that demographic characteristics, particularly age and gender, are consistently associated with the severity of these crashes (35, 37, 39, 40). For instance, prior research (35, 40) found that male VRUs are generally at a higher risk of experiencing severe injuries. In terms of age, previous studies have indicated that older VRUs (35, 39, 40) are more likely to sustain severe injuries, while crashes involving young and middle-aged drivers (35, 37) are more likely to result in severe injuries.

**Table 1:** Summary of the literature on heavy vehicle–VRU safety and injury severity.

Country/Dataset	Study context	Modeling technique	Key findings/risk factors	Literature
Norway/631	Examining conflicts between cyclists and trucks in metropolitan environments	Binomial and multinomial logistic regression	Truck overtaking bicyclist (↑), right-turning truck versus straight-riding bicyclist (↑), visibility factor (truck driver did not see a cyclist) (↑)	(43)
Australia/2,436	Investigating the impact of sociodemographic factors on the severity of injuries resulting from interactions between heavy vehicles and VRUs	Random parameters ordered logit model	Road users’ characteristics: Male (↑), age 65 or older (↑), VRUs (↑). Road users’ residential-neighborhood characteristics: Age 45–64 (↑), age 15–24 (↓), higher median incomes (↓), graduation-level education (↓), technical or trade education (↑), people working in sales or as laborers (↓). Crash-location neighborhood characteristics: Age 45–64 (↑), people working as laborers (↓), people working as machinery operators (↑), higher individual incomes (↓), higher household incomes (↑). Crash characteristics: Night (↑), weekdays (↑), speeding ≥60 km/h (↑), three or four lanes (↓), wider outer shoulder (↑)	(40)
United States/2,043	Analyzing the severity of injuries sustained by pedestrians and cyclists in incidents with heavy trucks	Random forest, decision tree	Local roads with AADT >38,000: Proximity to intersection (↑), vehicle speed (↑). High-volume roads not classified as local: Young/middle-aged truck drivers (↑), divided roadways (↑), speed limit of 50–75 mph (↑), midday period (↑). Low-volume roads: Clear weather (↑), combination of median barrier and curb shoulder close to a signalized intersection (↑)	(37)
United States/699 and 593	Exploring the safety of pedestrians and cyclists in collisions involving freight vehicles	Logit model, random parameters logit model	Vehicle-related factors: Small vehicles (↓), tractors with trailers (↑). VRU-related factors: Pedestrians (↑), age 55 years or older (↑), alcohol or drugs (↑). Driver-related factor: Alcohol or drugs (↑), right turn (↓). Time of day: 3:00–7:00 p.m. (↓). Crash environment: Dark–not lighted (↑), parking lots/private property (↓)	(39)
United States/23,090	Investigating the influence of heavy vehicle operations on the severity of injuries sustained by pedestrians and cyclists	Binomial logistic regression	Pickup truck (↑), single-unit truck (↑), van/minivan (↑), intersection (↓), children (↓), seniors (↑)	(14)
Canada/21	Improving interactions between heavy vehicle drivers and VRUs through driver training	Descriptive statistics	Truck maneuvers: Turns (↑), backing up (↑). VRU behavior: Crossing against a red light (↑), lack of respect or courtesy (↑), distraction (↑), presence of children (↑), pedestrian-dense roads (↑). Infrastructure: Lack of cycling lanes (↑), construction sites (↑), poorly designed intersections and traffic lights (↑). Weather: Inclement weather, including rain, snow, fog, and slush (↑)	(41)
South Korea/1,932	Examining factors influencing collisions between freight vehicles and non-motorized road users (pedestrians and cyclists)	Bayesian multivariate conditional autoregressive model	Distance to logistics terminals (↓), warehouse density (↑), neighborhoods with a denser network of non-highway roads (↑), higher population density (↓), higher employment density (↑), mixed land use (↑), bicycle traffic-volume density (↑), shared bicycle–pedestrian paths (↓)	(38)
China/2,037	Analyzing the severity of injuries resulting from crashes involving heavy vehicles and VRUs (pedestrians, cyclists, and electric-bike riders)	Random forest, stacking, extreme gradient boosting, voting	VRU age 57 or above (↑), truck driver age 30–37 (↑), male VRUs (↑), driving experience 13–16 years (↑), nighttime (↑), reduced visibility (↑), poor lighting conditions (↑), national roads (↑), provincial roads (↑), rural roads (↑)	(35)

Several studies have shown that temporal factors, such as the day of the week and time of day, are associated with injury severity in heavy vehicle–VRU crashes. For instance, existing research (40) reported that weekdays are associated with an increased risk of severe injuries in crashes involving VRUs and heavy vehicles. Previous studies have reported different patterns for time of day, with some identifying greater injury severity during daytime conditions (37), while others found a higher risk of severe outcomes at night (35, 39, 40). Associations have also been reported between weather conditions, including clear and inclement weather, and injury severity in collisions involving heavy vehicles and VRUs (37, 41). Moreover, darkness and inadequate roadway lighting have also been associated with greater injury severity (35, 39). Comparable evidence from broader VRU injury-severity research indicates that lighting conditions, road alignment, and physical separation can also be associated with the severity of electric-bike crashes (42).

Behavioral factors are another important group of variables associated with injury severity. The reported behavioral factors include speeding, distraction, traffic-rule violations, and unsafe interactions between heavy-vehicle drivers and VRUs. Prior research (37, 40, 41) identified speeding and distraction as important factors associated with the severity of injuries in these incidents. Existing research (41) also identified crossing against a red light as an important safety concern in interactions between heavy vehicles and VRUs. Previous research (39) reported that alcohol and drug use among drivers and VRUs is associated with a greater likelihood of severe crash outcomes.

The interaction between VRUs and various other vehicle categories, including small vehicles, vans/minivans, single-unit trucks, pickup trucks, and tractor-trailers, is also associated with differences in crash injury severity. Differences in size, weight, and design among interacting vehicles are associated with differences in injury outcomes (14, 39). For instance, existing work (39) concluded that VRUs interacting with smaller vehicles are less likely to sustain severe injuries, whereas encounters with larger and heavier vehicles, including tractor-trailers, are associated with a greater risk of serious injuries. Prior research (14) also found that collisions involving VRUs and pickup trucks, single-unit trucks, and vans/minivans are often associated with severe injury outcomes. Moreover, pedestrian involvement and the presence of children have also been associated with greater injury severity in some studies (39, 41).

Roadway geometric features, including functional class, design, lane width, outer shoulder dimensions, construction zones, and the presence of right turns and intersections, have been identified as important factors associated with injury severity in crashes involving heavy vehicles and VRUs (35, 37, 39–42). For instance, prior research (35) concluded that national, provincial, and rural roads are associated with an increased risk of severe injury crashes. Previous research (37, 40, 41) reported that divided roads, wider outer shoulders, the lack of bicycle lanes, and ongoing construction are associated with severe crash outcomes. Prior work (37, 41) also noted that turns, inadequately designed intersections, and proximity to intersections are associated with a higher injury severity. Previous studies (14, 38–40) observed that roads featuring three or four lanes, right turns, intersections, and shared bicycle–pedestrian lanes are associated with reduced crash severity.

### 2.2 Methodological approaches used in previous studies

Previous studies examining injury severity in crashes involving heavy vehicles and VRUs have employed a wide range of methodological approaches, as detailed in Table 1. For instance, existing research has employed descriptive and interview-based approaches to identify recurring interaction patterns, behavioral concerns, and infrastructure-related safety issues involving heavy vehicles and VRUs (41). While these approaches are useful for identifying recurring safety concerns and interaction patterns, they are limited in their ability to evaluate statistical associations or quantify the effects of explanatory variables.

Prior research has also employed conventional regression models to examine heavy vehicle–VRU safety and injury severity. For example, binomial and multinomial logistic regression have been used to examine cyclist–truck conflicts, while binomial logistic regression has been applied to pedestrian and pedalcyclist injury severity in crashes involving large vehicles (14, 43). These models allow the associations between observed crash characteristics and safety or injury outcomes to be estimated in an interpretable form. However, conventional fixed-parameter specifications assume that the estimated effects remain constant across observations, which may not adequately represent unobserved differences among heavy vehicle–VRU crashes (44).

Recent studies on heavy vehicle–VRU crash severity have also adopted machine learning methods, including random forest, decision tree, stacking, extreme gradient boosting, and voting (35, 37). Existing research has applied ensemble-learning methods to truck–VRU injury severity, while other studies have used random forest and decision-tree models to examine pedestrian and bicyclist crashes involving large trucks (35, 37). These methods can capture nonlinear relationships and complex interactions among crash characteristics. These methods, however, do not provide the same type of parameter-based inference as econometric approaches designed to estimate parameter effects and account explicitly for unobserved heterogeneity. The machine-learning studies summarized in Table 1 therefore provide an important complementary approach, but they do not address the same inferential objective as random-parameter models.

Within the heavy vehicle–VRU literature, random-parameter models have been introduced to account for unobserved heterogeneity across crashes. Prior research has applied a random parameters logit model to freight-vehicle crashes involving pedestrians and cyclists (39), while another study employed a random parameters ordered logit model to examine injury severity across multiple VRU groups involved in truck crashes (40). These approaches allow selected parameter effects to vary across observations and therefore relax the assumption that every crash is governed by the same fixed parameter values. However, the heavy vehicle–VRU studies identified in Table 1 do not explicitly model systematic heterogeneity in the means and variances of the random parameters. Consequently, these specifications do not explicitly identify whether observed crash characteristics systematically explain variation in the means or variances of the random-parameter distributions.

In this context, the present study employs a random parameters binary probit model with heterogeneity in means and variances. This approach allows model parameters to vary across observations while also allowing their means and variances to vary according to observed characteristics. Although this extension has not been identified in the heavy vehicle–VRU studies summarized in Table 1, random-parameter specifications that account for systematic heterogeneity in the means and/or variances of random parameters have been applied in broader injury-severity and VRU-safety research (22, 45–47). Prior applications indicate that observed characteristics can be incorporated to explain systematic variation in random-parameter distributions. The framework therefore provides a basis for examining not only whether parameter effects vary across heavy vehicle–VRU crashes, but also whether observed crash characteristics help explain that variation.

### 2.3 Study setting and data description

The research was carried out in Rawalpindi, located in the Punjab province of Pakistan. The city serves as the administrative center of the Rawalpindi Division and is adjacent to Pakistan’s capital, Islamabad. According to the most recent census report, Rawalpindi had a population of approximately 3.36 million in 2023, making it the fourth most populous city in Pakistan, after Karachi, Lahore, and Faisalabad (48). A detailed depiction of the study area is presented in Figure 1.

**Figure 1:**
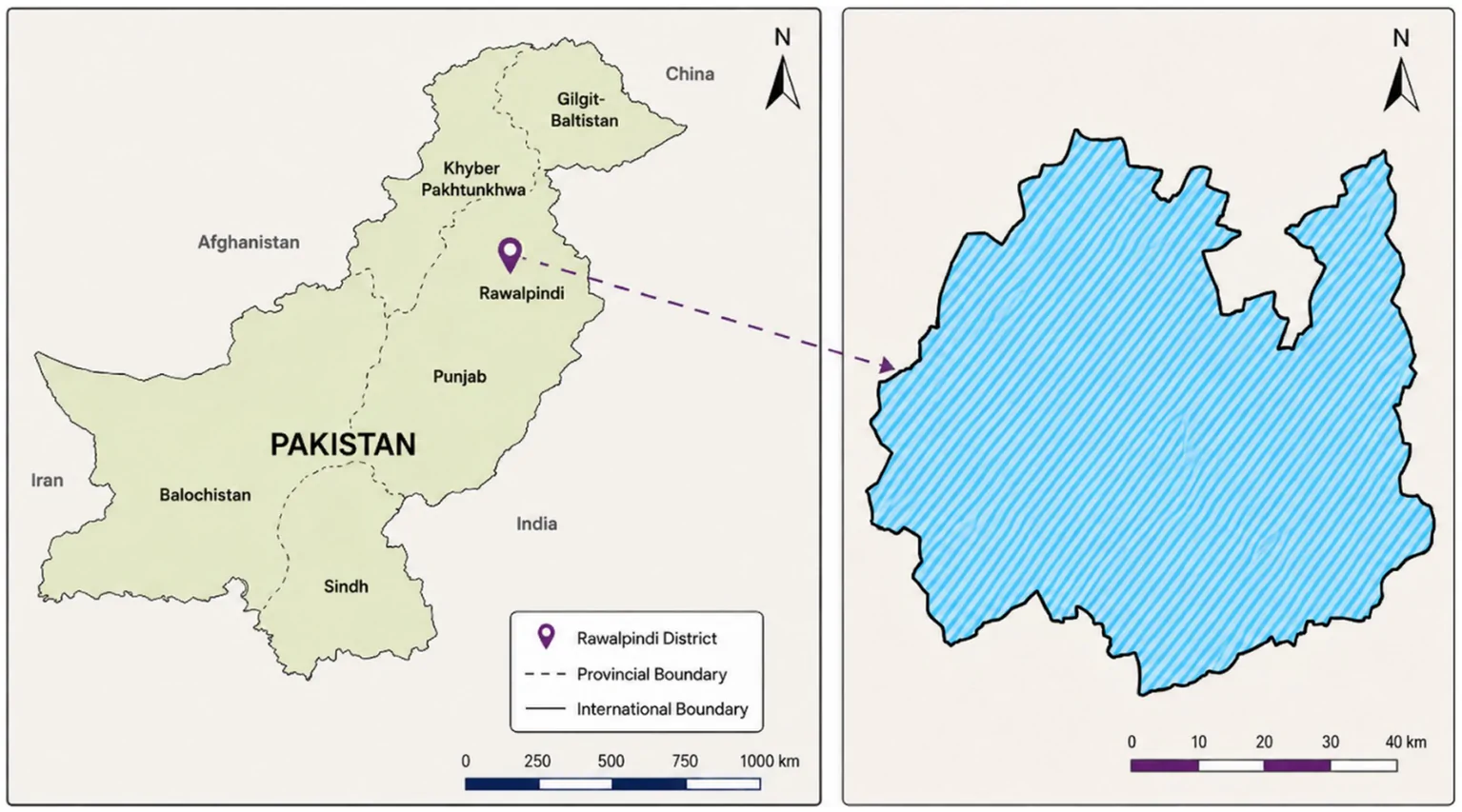
Map of the study area.

For the purposes of this study, heavy vehicles were operationally defined as heavy trucks and large buses recorded in the Rescue 1122 database and used for freight or passenger transportation, while the included VRUs comprised pedestrians, motorcyclists, and motorized rickshaw users. Crash records involving these heavy vehicles and VRUs were obtained from the Rescue 1122 office in Rawalpindi and covered the period from January 1, 2021, to July 31, 2023. Rescue 1122 is an emergency medical and rescue service responsible for responding to road traffic crashes and maintaining operational records of emergency incidents. The records were obtained with official permission for academic research purposes and were provided in anonymized form, with personally identifiable information removed before they were made available to the researchers.

The Rescue 1122 database contained information on road-user characteristics, crash temporal attributes, vehicle categories involved in the crashes, crash locations, and injury outcomes. Weather-related variables corresponding to each crash event were obtained from the Pakistan Meteorological Department in Islamabad. Roadway geometric characteristics were collected by the research team through field investigations at the reported crash locations. Prior to the field surveys, the assessors received training from the research team in the use of predefined criteria for identifying, measuring, and classifying roadway characteristics. The training was intended to ensure consistent evaluation of key roadway characteristics, including road alignment, lane configuration, lane width, shoulder width, median type, intersection characteristics, access points, and roadside facilities, across all crash locations. The information obtained from these sources was subsequently integrated and preprocessed to develop the analytical dataset. The integrated dataset initially contained 1,037 crash records, of which 30 (2.89%) had missing information in one or more variables required for the analysis. These records were excluded using listwise deletion, leaving a final dataset of 1,007 complete crash records.

Within the final analytical dataset, injury severity was classified into three categories: minor injuries (such as small and shallow cuts, abrasions, or bruises), serious injuries (such as skull, spine, or neck fractures, severe burns, or significant blood loss), and fatal injuries. Among the 1,007 records, 232 (23.04%) were classified as minor injuries, 747 (74.18%) as serious injuries, and 28 (2.78%) as fatal injuries. Given that fatal injuries accounted for only 2.78% of the sample (n = 28), fatal and serious injuries were combined to form a single “severe injury” category, while minor injuries were categorized as “non-severe injuries.” Consequently, 775 (76.96%) cases were classified as severe, and 232 (23.04%) were classified as non-severe. Similar binary classifications of injury severity have been widely used in previous studies to investigate injury severity outcomes among VRUs (23, 39). A detailed summary of the descriptive analysis is provided in Table 2. For model estimation, categorical variables were represented using indicator variables, with one category omitted as the reference category.

**Table 2:** Operational definitions and descriptive statistics.

Variable	Operational definition	Category	Share (%)
Injury severity level	Injury outcome sustained by the VRU in the crash	Severe	76.96
		Non-severe	23.04
VRU category	Type of VRU involved in the crash	Pedestrian	18.77
		Motorcyclist	72.09
		Motorized rickshaw user	9.14
VRUs’ gender	Gender of the VRU	Male	89.28
		Female	10.72
VRUs’ age group	Age group of the VRU at the time of the crash	≤ 25	42.11
		26–40	32.17
		41–50	12.61
		> 50	13.11
Season	Season during which the crash occurred	Spring	26.41
		Summer	30.69
		Autumn	14.80
		Winter	28.10
Day of the week	Whether the crash occurred on a weekday or weekend	Weekday	72.19
		Weekend	27.81
Period of the day	Traffic demand period when the crash occurred. Peak refers to morning/evening rush hours; off-peak includes all other periods	Peak hour	23.24
		Off-peak hour	76.76
Time of day	Time period when the crash occurred	Daytime	61.07
		Nighttime	38.93
Weather condition	Weather condition at the time of the crash	Clear	63.16
		Cloudy	29.00
		Rainy	7.84
Recorded contributing factor	Primary contributing factor recorded for the crash	Speeding	63.75
		Distraction	30.69
		Other	5.56
Road functional class	Functional classification of the roadway	Primary	92.45
		Secondary	5.86
		Tertiary	1.69
Number of lanes	Number of traffic lanes in the direction of travel at the crash location	One	7.45
		Two	37.83
		Three	14.20
		Four	36.35
		Five	4.17
Lane width (m)	Width of an individual traffic lane measured during the field survey	≥ 3.5	24.13
		< 3.5	75.87
Median type	Type of median separating opposing traffic streams	Grassy	73.29
		New Jersey barrier	14.30
		Painted	12.41
Outer shoulder width (m)	Width of the outer shoulder measured during the field survey	≥ 1.5	42.20
		< 1.5	57.80
Alignment type	Roadway alignment at the crash location	Straight	59.78
		Horizontal curve	30.78
		Vertical curve	9.44
U-turn	Presence of a U-turn facility at the crash location	Yes	61.87
		No	38.13
Intersection	Whether the crash occurred at or within the influence area of an intersection	Yes	26.32
		No	73.68
Access point	Presence of a driveway or property access point at the crash location	Yes	80.24
		No	19.76
Sidewalk	Presence of a pedestrian sidewalk at the crash location	Yes	31.28
		No	68.72

The descriptive statistics show that motorcyclists represented the largest VRU group in the crash records, accounting for 72.09% of the records, followed by pedestrians (18.77%) and motorized rickshaw users (9.14%). Crashes involving male VRUs accounted for 89.28% of the cases. VRUs aged 25 years or younger represented the largest age group (42.11%), followed by those aged 26 to 40 years (32.17%). The records further show that 30.69% of the crashes occurred during the summer season, 72.19% on weekdays, 61.07% during the daytime, and 63.16% under clear weather conditions. Speeding and distraction were recorded as contributing factors in 63.75 and 30.69% of the crashes, respectively. Most crashes occurred on primary roads (92.45%), while 59.78% occurred along straight road segments and 73.29% at locations with grassy medians. Narrow lanes with widths below 3.5 m and outer shoulders with widths below 1.5 m were present in 75.87 and 57.80% of the crashes, respectively. Other frequently recorded roadway characteristics included the presence of U-turns (61.87%) and access points (80.24%), while 73.68% of the crashes occurred at non-intersection locations and 68.72% occurred at locations without sidewalks.

## 3 Methodology

### 3.1 Analytical approach

This study examines the factors associated with injury severity in crashes involving heavy vehicles and VRUs, where the dependent variable distinguishes between severe and non-severe injury outcomes. Given the binary structure of this response variable, a binary probit model was selected as an appropriate analytical tool, consistent with probit-based approaches used in injury-severity research (49, 50). Therefore, in the conventional binary probit model, severe injury outcomes were designated as $Y_{i}=1$ and non-severe outcomes as $Y_{i}=0$ . The probability of a severe injury for the i th observation can be expressed as follows (49):


$$P(Y_{i}=1)=\int_{-\infty }^{\beta_{0}+\sum_{k=1}^{K}\beta_{k}X_{\mathrm{ik}}}\frac{1}{\sqrt{2\pi }}\exp(-\frac{t^{2}}{2}\;)\mathrm{dt}$$

In the above equation, $P(Y_{i}=1)$ represents the probability of a severe injury outcome, the integral corresponds to the cumulative distribution function of the standard normal distribution, $\beta_{0}$ denotes the intercept, and $\beta_{k}$ represents the fixed coefficient corresponding to the independent variable $X_{\mathrm{ik}}$ .

In this foundational model, all parameters are treated as constant across observations, implying that each factor’s effect on the probability of a severe injury outcome remains uniform. Although this assumption simplifies analysis, it may fail to capture variation across crashes, potentially leading to biased estimates due to unobserved heterogeneity (51). For example, in the present study context, unobserved factors like a pedestrian’s reaction time to an approaching truck or the driver’s fatigue level, neither of which was included in the dataset, may help explain variation in injury severity across crashes. To overcome this limitation, the basic model is extended by allowing certain parameters to vary across observations, allowing the model to account for unobserved heterogeneity across crashes (45):


$$P(Y_{i}=1)=\int \Phi (\beta_{0}+\sum_{k=1}^{K}\beta_{k}X_{\mathrm{ik}}\;)f(\beta |\phi )\mathrm{d\beta}$$

In the above equation, f(β∣φ) represents the density function of the random parameter vector β and φ denotes the corresponding distributional parameters. However, this initial extension treats the means and variances of the random parameters as constant across observations, and therefore does not account for systematic variation in these distributional properties (52). To better account for possible variations in the means and variances of the random parameters, these distributional properties can be specified as functions of observed factors. This refinement leads to the random parameters binary probit model with heterogeneity in means and variances. The k th random parameter for observation i can be expressed as follows (53):


$$\beta_{\mathrm{ki}}=\beta_{k}+\gamma_{k}Z_{i}+\sigma_{k}\exp(w_{k}W_{i})v_{\mathrm{ki}}$$

In the above equation, $\beta_{k}$ is the mean value of the k th parameter across all crashes, and $Z_{i}$ represents a set of explanatory variables affecting the mean of the random parameter with corresponding coefficients $\gamma_{k}$ . $W_{i}$ is a vector of variables capturing heterogeneity in the standard deviation $\sigma_{k}$ with corresponding coefficients $w_{k}$ , and $v_{\mathrm{ki}}$ is a random error term.

In the present research, model estimation was conducted using simulated maximum likelihood with 1,000 Halton draws to improve simulation accuracy (54). The statistical analysis was carried out using NLOGIT 5 software. To determine the most appropriate distribution for the random parameters, multiple distributions were evaluated, and the normal distribution was found to be the most suitable, aligning with prior research (51, 55–57).

In the current research, model performance was assessed using the Akaike Information Criterion (AIC), which balances model fit against complexity (58). The Bayesian Information Criterion (BIC) was also used as a complementary measure, with its penalty for model complexity increasing with sample size (59). McFadden’s $\rho^{2}$ , a commonly used pseudo- $R^{2}$ measure for discrete-choice models, was calculated (60). Marginal effects were calculated to quantify changes in the predicted probability of a severe injury outcome. For continuous variables, the marginal effect represents the change associated with a one-unit increase in the variable, while for indicator variables it represents the discrete change associated with a shift from 0 to 1 (49).

### 3.2 Model validation and refinement

To assess potential multicollinearity among explanatory variables, variance inflation factors (VIFs) were calculated. The analysis showed that the highest VIF value was 3.62, with the largest values corresponding to the variables “recorded contributing factor (other)” and “intersection presence.” This result suggests that no serious multicollinearity was present among the explanatory variables included in the model. Following this assessment, the initial candidate variables were identified based on their relevance to injury severity and evidence from previous crash-severity studies. Statistical significance at the 10% level or stricter was then used as an additional criterion during model refinement. Likelihood ratio tests were subsequently used to determine whether the more flexible model specifications provided statistically significant improvements in fit relative to the corresponding nested models.

Within the random parameters binary probit model, parameters showing statistically significant standard deviations were treated as random parameters, while those without significant variation were classified as fixed parameters. After estimating this model, further exploration was carried out to examine heterogeneity in the means and variances of the random parameters. This step involved systematically testing each statistically significant explanatory variable to determine its potential impact on the distributional properties, specifically the means and variances, of the random parameters in the refined random parameters binary probit model with heterogeneity in means and variances. This detailed investigation sought to reveal underlying patterns or relationships that might account for the variability observed in the effects of explanatory variables across crashes, thereby enhancing the model’s ability to reflect real-world complexities and ensuring a comprehensive understanding of how explanatory factors influence the probability of severe injury.

## 4 Results and discussion

### 4.1 Model estimation results

This study applied the random parameters binary probit model with heterogeneity in means and variances to investigate factors associated with crash injury severity in incidents involving heavy vehicles and VRUs. To compare model performance, three models were estimated: the basic probit model, the random parameters probit model, and the random parameters probit model with heterogeneity in means and variances. Table 3 summarizes the goodness-of-fit statistics for these competing models, providing a clear basis for evaluating their performance. The three models were developed using 1,007 observations, and the analysis progressively increased in complexity, estimating 20 parameters for the basic model, 24 for the random parameters probit model, and 29 for the random parameters probit model with heterogeneity in means and variances, reflecting the additional flexibility in capturing variability across crashes.

**Table 3:** Goodness-of-fit statistics for the competing models.

Estimated model statistics	BPM	RPPM	RPPM_HMV
Observations	1,007	1,007	1,007
Parameters estimated	20	24	29
Log-likelihood at convergence ( $LL_{\beta }$ )	−513.340	−499.089	−481.062
Log-likelihood at constants ( $LL_{0}$ )	−543.522	−543.522	−543.522
AIC	1066.680	1046.178	1020.124
BIC	1165.000	1164.162	1162.688
McFadden $\rho^{2}$	0.056	0.082	0.115
Likelihood ratio test	RPPM vs. BPM	RPPM_HMV vs. RPPM	
$\chi^{2}=-2[LL_{\text{simple model}}-LL_{\text{complex model}}]$	28.502	36.054	
Degrees of freedom	4	5	
*p*-value	< 0.001	< 0.001	

BPM, Basic Probit Model; RPPM, Random Parameters Probit Model; RPPM_HMV, Random Parameters Probit Model with Heterogeneity in Means and Variances.

The results indicate that the random parameters probit model with heterogeneity in means and variances provides a better fit than both simpler alternatives. Specifically, the log-likelihood at convergence improves from −513.340 in the basic probit model to −499.089 in the random parameters probit model, and further to −481.062 in the random parameters probit model with heterogeneity in means and variances. This trend indicates a better fit as the model accounts for random parameter variation and heterogeneity in their means and variances. Similarly, the AIC decreases from 1066.680 (basic probit model) to 1046.178 (random parameters probit model), and down to 1020.124 (random parameters probit model with heterogeneity in means and variances), while the BIC drops from 1165.000 to 1164.162, and slightly further to 1162.688 across the three models. The AIC and BIC values were lowest for the random parameters probit model with heterogeneity in means and variances, suggesting a more favorable balance between model fit and complexity among the three specifications.

In addition, McFadden’s $\rho^{2}$ increased across the three specifications, from 0.056 in the basic probit model to 0.082 in the random parameters probit model and 0.115 in the model with heterogeneity in means and variances. Moreover, to further assess whether the increase in model complexity led to statistically meaningful improvements in fit, likelihood ratio tests were conducted. Comparing the random parameters probit model to the basic probit model yielded a chi-square value of 28.502 with 4 degrees of freedom and a *p*-value less than 0.001, confirming that adding random parameters significantly improves model fit. Similarly, comparing the random parameters probit model with heterogeneity in means and variances to the random parameters probit model produced a chi-square value of 36.054 with 5 degrees of freedom and a *p*-value below 0.001, providing evidence that accounting for heterogeneity in means and variances further improves the model. Accordingly, the final model was selected based on the combined evidence from the log-likelihood, AIC, BIC, McFadden’s $\rho^{2}$ , and likelihood-ratio tests.

Based on these model-comparison results, the results presented in Table 4 and discussed in the subsequent sections are based on the random parameters probit model with heterogeneity in means and variances. This choice allows the analysis to capture both the variability in risk factors and their underlying heterogeneity, offering a more nuanced understanding of crash severity dynamics. In addition, Table 4 presents the marginal effects derived from the random parameters probit model with heterogeneity in means and variances, quantifying changes in the predicted probability of severe injury outcomes associated with the explanatory variables.

**Table 4:** Modeling results.

Variable	Coefficient	Std. error	*p*-value	Marginal effects (%)
Constant	0.437**	0.221	0.048	–
Fixed parameters				
VRUs’ gender				
Male	- 0.492***	0.169	0.003	−13.253
Female	Reference			
VRUs’ age				
≤ 25	0.434***	0.106	< 0.001	7.774
26–40	Reference			
41–50	0.372	0.234	0.112	–
> 50	0.418	0.265	0.115	–
Day of week				
Weekday	Reference			
Weekend	0.225**	0.102	0.027	4.569
Weather conditions				
Clear	Reference			
Cloudy	0.286	0.182	0.116	
Rainy	0.252**	0.116	0.030	8.019
VRUs’ type				
Pedestrian	0.577***	0.184	0.002	15.289
Motorcyclist	0.472***	0.148	0.001	13.071
Motorized rickshaw user	Reference			
Road class				
Primary	0.307*	0.185	0.096	4.961
Secondary	0.453	0.281	0.107	
Tertiary	Reference			
Number of lanes				
One lane	0.347	0.219	0.113	
Two lanes	0.476***	0.107	< 0.001	12.400
Three lanes	0.282**	0.142	0.047	7.374
Four lanes	0.294	0.187	0.116	
Five lanes	Reference			
Road alignment				
Straight	Reference			
Horizontal curve	0.431	0.268	0.108	
Vertical curve	0.619***	0.215	0.004	16.821
Sidewalk				
Yes	0.296***	0.102	0.003	8.053
No	Reference			
Access point				
Yes	0.253***	0.096	0.008	3.388
No	Reference			
Random parameters				
Nighttime	0.352***	0.104	< 0.001	7.318
Std. dev.	0.462***	0.087	< 0.001	-
Distraction	0.411***	0.122	< 0.001	16.877
Std. dev.	0.450***	0.101	< 0.001	–
Heterogeneity in means				
Nighttime – Pedestrian	0.526*	0.291	0.071	–
Nighttime – Access point (yes)	0.447*	0.229	0.051	–
Nighttime – Rainy	0.337**	0.148	0.023	–
Distraction – Pedestrian	0.591**	0.297	0.046	
Heterogeneity in variance				
Nighttime – Pedestrian	−0.376*	0.220	0.087	–

*p < 0.10; **p < 0.05; ***p < 0.01. Reference categories are indicated in the table.

### 4.2 VRUs’ attributes

The analysis indicates that crashes involving heavy vehicles and male VRUs are less likely to result in severe injury outcomes compared to female VRUs. Specifically, the marginal effect for male VRUs shows a 13.253% decrease in the probability of severe injury relative to female VRUs. One possible explanation for this association relates to the physiological differences between male and female VRUs. Prior research (61–63) has documented gender-related differences in crash injury and fatality risk, suggesting that physiological and biomechanical differences may contribute to the observed pattern. In addition, behavioral differences may also contribute to this pattern. For instance, studies (64, 65) suggest that male VRUs often demonstrate faster reaction times and better situational awareness in traffic situations, which may help them avoid direct collisions or reduce the severity of impact.

The findings further show that age is an important factor in crashes involving heavy vehicles and VRUs, with younger VRUs (aged ≤ 25) being more likely to experience severe injury outcomes. The marginal effect indicates that this group is associated with a 7.774% higher probability of sustaining severe injuries compared to middle-aged VRUs (aged 26–40). This finding may reflect a combination of limited traffic experience and a greater tendency toward risky road behavior among younger road users. Prior research suggests that younger VRUs may be less capable of accurately judging the speed, stopping distance, and movement characteristics of heavy vehicles, which may increase their vulnerability in conflict situations (66, 67). In addition, younger individuals are more likely to engage in behaviors such as jaywalking, abrupt lane changes, or ignoring traffic signals, all of which may increase the probability of severe injury when interacting with heavy vehicles (68–70).

### 4.3 Temporal characteristics

Crashes involving VRUs and heavy vehicles that occur during weekends were associated with a 4.569% higher probability of severe injury than crashes occurring on weekdays. This pattern may reflect a combination of behavioral, environmental, and emergency response conditions that distinguish weekend travel from weekday travel. Prior research on weekend crashes has identified several driver-related risk factors, including fatigue, impaired driving, and non-use of seatbelts, that are associated with severe injury outcomes (71). In crashes involving heavy vehicles, these behaviors may have more serious consequences for VRUs, who lack protective structures and are therefore more vulnerable to direct impact forces (23, 72). Another possible explanation relates to post-crash conditions. In some settings, weekends are associated with delays in emergency medical response and reduced access to timely trauma care, both of which may worsen injury outcomes after a collision has occurred (73, 74). In low- and middle-income countries, this relationship may be further influenced by mixed traffic environments, characterized by the close interaction of heavy vehicles with VRUs, limited lane discipline, and frequent risky overtaking, all of which may increase the probability of severe-injury crashes (31, 32). In settings such as Pakistan, where motorcyclists, pedestrians, motorized rickshaws, and heavy vehicles often operate in close proximity, these weekend conditions may leave VRUs with less time and space to avoid serious conflicts.

The results indicate that nighttime conditions are associated with a 7.318% increase in the probability of severe injury outcomes in crashes involving heavy vehicles and VRUs. Nighttime driving is commonly associated with behavioral and environmental risk factors, including driver fatigue, reduced alertness, and higher operating speeds when traffic volumes are lower. These conditions may reduce the ability of drivers to detect and respond to VRUs in a timely manner, particularly when VRUs are less visible or are moving along poorly illuminated road edges. In the case of heavy vehicles, delayed detection is especially important, as their larger blind spots, wider turning paths, and longer stopping distances leave less room for corrective action once a conflict emerges (75). In low- and middle-income countries, the greater severity of nighttime crashes may also be related to inadequate roadway lighting, weak enforcement of traffic regulations, and limited separation between motorized traffic and VRUs (26, 42). In addition, the presence of street vendors and pedestrians near the roadway may create additional conflicts, potentially increasing the risk of collisions and severe injuries (29, 76). These factors are particularly relevant in mixed-traffic environments such as Pakistan, where heavy vehicles, motorcyclists, pedestrians, motorized rickshaws, and roadside activities often operate in close proximity, which may increase the likelihood of nighttime conflicts and more severe injury outcomes.

### 4.4 Weather conditions

The findings indicate that rainy weather is associated with an 8.019% increase in the probability of severe injury outcomes in crashes involving heavy vehicles and VRUs. During rainy conditions, reduced tire–road friction and longer braking distances may impair the maneuverability and stopping performance of heavy vehicles, whose large mass and operational limitations already make them less responsive in conflict situations. This, in turn, may increase the likelihood of high-impact collisions when sudden conflicts with VRUs occur, potentially contributing to more severe injury outcomes (41, 77). Moreover, rainfall may also reduce visibility, making it more difficult for drivers to detect VRUs in time, particularly in urban environments with poor lighting and visually complex traffic conditions (35, 77). Road-user behavior may further contribute to this relationship. Under rainy conditions, unsafe road-user behaviors, including traffic-rule violations and risky driving, may reduce the ability of both drivers and VRUs to respond safely to developing conflicts (41, 78). In Pakistan, these risks are also associated with infrastructural deficiencies, such as inadequate drainage systems, which can contribute to water accumulation on road surfaces and may increase the likelihood of skidding and loss of vehicle control (77). In addition, in the country’s mixed-traffic environment, where heavy vehicles often operate close to pedestrians, motorcyclists, and motorized rickshaws, these roadway conditions may leave limited room for evasive action once a conflict begins to develop.

### 4.5 Crash attributes

The results demonstrate that among VRUs, pedestrians and motorcyclists are associated with a 15.289 and 13.071% higher likelihood of severe injury, respectively, in crashes involving heavy vehicles. This result may reflect the strong mismatch between the physical vulnerability of these road users and the large size and mass of heavy vehicles, all of which are associated with more serious injury outcomes during collisions (39, 77). Pedestrians show the greatest increase in severe injury probability, which may be related to the complete lack of physical protection at the time of collision, leaving them directly exposed to high-energy impacts (14, 39). Previous research has linked inadequate pedestrian infrastructure, including access points, intersections, and insufficient VRU facilities, with greater pedestrian injury risk and safety concerns in interactions with heavy vehicles (23, 41). Motorcyclists also remain highly vulnerable in collisions with heavy vehicles (21). The large difference in vehicle size and operating characteristics may place motorcyclists at risk during close interactions with heavy vehicles (21, 41). These findings are consistent with prior studies, which indicate that collisions involving heavy vehicles and pedestrians or motorcyclists are associated with a substantially higher risk of severe injury outcomes (21, 23, 39).

The findings indicate that distraction is associated with a 16.877% increase in the probability of severe injury outcomes in collisions involving heavy vehicles and VRUs. VRUs often operate in close proximity to heavy vehicles and have limited physical protection; when they are distracted, their ability to perceive hazards, judge vehicle speeds, and execute evasive maneuvers may be reduced. This reduction in situational awareness is especially important in interactions with heavy vehicles, where safe movement often depends on timely judgments about speed, distance, turning behavior, and lane position. Once attention is diverted, VRUs may enter conflict zones, misjudge vehicle approach, or fail to respond quickly enough to avoid impact. In the case of heavy vehicles, the opportunity for conflict avoidance is already limited by their longer stopping distances, larger blind spots, and wider turning paths, which may help explain why distraction is associated with more severe injury outcomes (75). This finding aligns with existing road safety literature, which identifies VRU distraction as an important factor associated with injury severity, especially in mixed-traffic conditions common in low- and middle-income countries (79, 80). In such settings, inadequate enforcement, complex traffic flows, and limited VRU infrastructure may further worsen the safety consequences of VRU distraction (31, 32, 80).

### 4.6 Geometric features

The analysis showed that crashes involving heavy vehicles and VRUs on primary roads are associated with a 4.961% higher probability of severe injury outcomes. Primary roads typically carry higher traffic volumes and allow greater operating speeds, which substantially increase the kinetic energy generated during collisions (81). When conflicts occur between heavy vehicles and VRUs under such conditions, the resulting impact forces are substantially greater, and higher impact forces are associated with more severe injury outcomes (81). In addition, multi-lane primary roads are also often characterized by frequent lane-changing and risky overtaking maneuvers, which may increase conflict opportunities and leave VRUs with less space and time to avoid fast-moving traffic (31, 32). In many low- and middle-income country contexts, including Pakistan, primary roads often lack adequate pedestrian infrastructure, effective traffic calming measures, and proper access management. Previous studies have associated these deficiencies with greater VRU exposure, more frequent conflicts with heavy vehicles, and severe injury outcomes (23, 77). In the context of Pakistan, primary roads function not only as high-speed corridors but also as locations where VRUs remain exposed to heavy-vehicle traffic under conditions that are not forgiving once a conflict occurs.

The results indicate that crashes involving heavy vehicles and VRUs on two- and three-lane roads are associated with 12.400 and 7.374% increases in the likelihood of severe injury outcomes, respectively. Two-lane roads frequently lack dedicated facilities for VRUs, so VRUs often share limited roadway space with heavy vehicles. Previous research has associated such shared-space conditions with direct conflicts and severe-injury crashes (41). Three-lane roads provide additional capacity compared to two-lane facilities, but they still pose considerable safety challenges for VRUs, particularly in environments characterized by weak lane discipline and inadequate enforcement of traffic regulations (31, 32). Under these conditions, the extra lane does not necessarily improve safety, as irregular lane use, overtaking behavior, and encroachment into shared road space may still place VRUs at considerable risk. Narrow lanes and limited shoulder widths may add to this problem by reducing the space available for corrective maneuvers or evasive action once a conflict begins to develop (82, 83). Consistent with these findings, prior research conducted in the same context has also identified two- and three-lane roads as high-risk environments for severe injury crashes involving VRUs (72, 84).

The findings highlight vertical alignment as a critical geometric risk factor, with crashes on vertical curves associated with a 16.821% higher probability of severe injury outcomes in crashes involving heavy vehicles and VRUs. Vertical curves can reduce available sight distance, limiting drivers’ ability to perceive and respond to VRUs in a timely manner, particularly in mixed-traffic environments where VRUs frequently operate in close proximity to heavy vehicles (72, 85). For heavy vehicles, the adverse safety effects of vertical curves are compounded by their substantial mass, extended stopping distances, and limited acceleration and deceleration capabilities. As a result, when a heavy vehicle encounters a vertical curve, restricted visibility combined with delayed braking responses may increase the likelihood of high-impact collisions following sudden conflicts with VRUs, contributing to severe injury outcomes (85, 86). In Pakistan, these risks may also be compounded by roadway design limitations and limited VRU facilities (23, 72). This finding suggests that vertical curves are not only a visibility issue, but also a location where the operating limitations of heavy vehicles and the exposure of VRUs intersect in a particularly unsafe way.

The analysis indicates that the presence of sidewalks is associated with an 8.053% higher probability of severe injury outcomes in crashes involving heavy vehicles and VRUs. This seemingly counterintuitive association should be interpreted cautiously, as it does not imply that sidewalks themselves increase injury severity. One possible explanation is that sidewalk presence may capture characteristics of locations with greater pedestrian exposure and more demanding traffic conditions. Previous research in a low- and middle-income setting has shown that severe pedestrian crashes are concentrated on high-speed, high-traffic-volume, multilane roads with substantial pedestrian activity (87). When heavy vehicles are involved in these interactions, the severity of the resulting injuries may be greater given their larger mass and longer stopping distances (23, 75). The observed association may therefore reflect differences in pedestrian exposure and roadway context at locations where sidewalks are present, rather than a harmful effect of sidewalks themselves.

The results show that the presence of an access point is associated with a 3.388% increase in the probability of severe injury outcomes in crashes involving heavy vehicles and VRUs. One possible explanation for this association is that access points often create complex traffic conditions, generating frequent vehicle entry and exit movements. These situations pose a particular risk to VRUs, who may be less visible, especially when heavy vehicles are involved, as they are typically associated with large blind spots and limited maneuverability (75, 88). In low- and middle-income countries such as Pakistan, these risks may be compounded by aggressive driving behaviors, limited enforcement of traffic regulations, and inadequate road infrastructure (89). A prior study conducted in the same context also reported that proximity to access points significantly increases the likelihood of severe injury crashes involving VRUs (23). In practical terms, access points introduce localized instability into the traffic stream, and this instability may be especially dangerous for VRUs when heavy vehicles are entering, exiting, or turning within confined spaces.

### 4.7 Random parameters and their heterogeneity in the means and variances

All statistically significant explanatory variables were examined to determine whether they explained heterogeneity in the means and variances of the random parameters. The modeling results show that nighttime and distraction were random parameters. Nighttime exhibited heterogeneity in both its mean and variance, whereas distraction exhibited statistically significant heterogeneity in its mean only.

Among these two random parameters, nighttime had a baseline mean of 0.352 and a baseline standard deviation of 0.462, with both its mean and variance changing according to the heterogeneity terms included in the model. A plausible explanation is that the nighttime effect depends strongly on crash-specific conditions, including roadway lighting, roadside activity, traffic composition, and the degree of separation between heavy vehicles and VRUs (29, 31, 32, 90). In mixed-traffic settings such as Pakistan, these conditions can vary considerably from one road environment to another, which helps explain why the nighttime parameter is not fixed. In addition, the heterogeneity-in-means results show that the effect of nighttime becomes stronger in specific contexts. In particular, the mean of the nighttime parameter increases in crashes involving pedestrians, at locations with an access point, and under rainy weather conditions (23, 72, 91). These results indicate that the nighttime effect is not uniform across crashes and becomes stronger when the crash context already contains conditions that reduce visibility, complicate traffic movements, or increase VRU exposure. The stronger nighttime effect for pedestrians may reflect their greater vulnerability after dark, especially in locations where walking occurs close to the carriageway or where pedestrian facilities are limited (91). The stronger effect at access points may be related to the added complexity created by vehicle entry, exit, and turning movements, which become more difficult to interpret and respond to at night. Likewise, the stronger nighttime effect under rainy conditions indicates that darkness and rain together may create especially unsafe conflict conditions for VRUs interacting with heavy vehicles. The model also identifies heterogeneity in the variance of the nighttime parameter through the interaction with pedestrians. The negative coefficient for this term indicates that the variability in the nighttime effect becomes smaller when a pedestrian is involved, meaning that the effect is more consistent across pedestrian-related crashes.

The results also identify distraction as a random parameter. The estimated baseline mean and standard deviation are 0.411 and 0.450, respectively. The statistically significant standard deviation indicates that its effect varies across crashes, while the heterogeneity-in-means result shows that its mean changes with pedestrian involvement, consistent with previous studies on distraction-affected crashes (80, 92). One possible explanation for this variation is that the consequences of distraction may depend on factors such as traffic conditions, road layout, and the surrounding built environment (92). The heterogeneity-in-means term for distraction-pedestrian is positive and statistically significant, showing that the effect of distraction becomes stronger when the involved VRU is a pedestrian. This result indicates a stronger association between distraction and severe injury in pedestrian-related crashes involving heavy vehicles. Compared with other VRUs, pedestrians depend heavily on visual attention, movement judgment, and timely decision-making to avoid conflicts (80). Once attention is diverted, the opportunity to avoid a heavy vehicle may be severely limited, particularly in mixed-traffic environments where pedestrians often move close to vehicle streams with minimal physical separation.

### 4.8 Policy implications

The findings of this study highlight the need for more targeted safety policies to reduce severe injury outcomes in crashes involving heavy vehicles and VRUs, particularly in Pakistan and other low- and middle-income countries with mixed traffic conditions. A central result of this study is that pedestrians and motorcyclists face a substantially higher probability of severe injury in collisions with heavy vehicles. This is not simply a matter of general road-safety awareness; it is a matter of corridor design, freight operations, and vehicle safety. International practice addresses heavy–VRU crash risk through a combination of safer heavy-vehicle design and stronger separation of exposed road users from truck traffic. For example, the European Union has long required side underrun protection on heavy goods vehicles to reduce the risk of pedestrians and motorcyclists being pulled under heavy vehicles (93), while London’s Direct Vision Standard and Heavy Goods Vehicle Safety Permit Scheme link heavy-vehicle operation to driver visibility and required safety equipment (94). These examples suggest that Pakistan should move beyond generic safety messaging and prioritize truck-intensive corridors for pedestrian protection, reduction of conflicts involving motorcyclists, and safer heavy-vehicle operating requirements. In addition to engineering measures, road authorities should intensify speed management, lane-discipline control, and overtaking enforcement in areas with high pedestrian and motorcyclist activity. Moreover, from an education perspective, public safety campaigns should explain the specific risks of interacting with heavy vehicles, including blind spots, wide turning paths, and limited stopping capability, rather than relying only on general traffic-safety messages (75).

Another important implication follows from the higher severe-injury risk observed among younger VRUs. This result suggests that Pakistan needs age-targeted safety measures rather than relying only on broad public awareness campaigns. A practical direction is to integrate heavy-vehicle interaction risk into school-based road-safety education, two-wheeler and, particularly, motorcycle licensing curricula, and novice-rider training. International guidance already supports this kind of staged and age-sensitive approach. WHO’s powered two- and three-wheeler safety manual discusses graduated licensing as a recognized tool for managing novice rider risk, and international transport safety work has similarly highlighted the value of graduated systems for novice and younger riders (95, 96). In practical terms, Pakistan could adapt this approach by introducing structured modules for younger riders and students on blind spots, truck turning paths, stopping distances, and safe positioning around heavy vehicles, while linking these modules to licensing and school-based safety education. In addition, enforcement should complement these programs through targeted monitoring around schools, colleges, vocational centers, and corridors with high concentrations of younger road users, where two- and three-wheelers and freight traffic operate in close proximity.

Among the roadway findings, vertical curves and access points deserve especially strong policy attention because they represent concrete locations where intervention is feasible. For vertical curves, the main issue is not only geometry in an abstract sense, but the combination of restricted sight distance, heavy-vehicle operating limitations, and VRU exposure. Research on vertical-curve safety has shown that perceptual treatments can reduce operating speed on crest-curve sections (97), which supports the use of targeted warning signs, reflective delineation, lighting, and localized speed control where heavy vehicles and VRUs interact. In practical terms, Pakistan should identify vertical-curve locations on roads with substantial truck movement and subject them to site-specific safety audits focused on sight distance, warning provision, visibility, speed management, and nearby pedestrian or two-wheeler activity. Access points require a similarly concrete response. Research on access management has shown that crash risk increases where access density and uncontrolled turning movements are high, while safety can be improved through turn-lane provision, median control, conflict-point reduction, and the separation of turning traffic from through traffic (88, 98). For Pakistan, this means that roads with frequent truck movements should be reviewed for unmanaged access points, especially where pedestrians and two-wheelers mix with entering, exiting, or turning freight traffic. In addition to engineering treatments, enforcement should focus on illegal turning, unsafe entry and exit movements, and roadside encroachment near these locations, while education efforts should warn local road users about the specific risks created by truck turning paths and limited visibility at access points and crest-curve sections.

The findings for nighttime suggest that enforcement and operational policy should also become more selective and context-based. The model indicates that the effect of nighttime is not uniform but varies across crash contexts and becomes more problematic in certain environments. A practical implication is that Pakistan should move toward targeted nighttime risk management instead of relying only on daytime enforcement or broad citywide policing. Research and guidance on nighttime pedestrian safety have shown that lighting quality and visibility conditions are important considerations for after-dark pedestrian safety (99–101). These findings support the use of intersection lighting, crosswalk visibility enhancements, reflective delineation, and improved pedestrian conspicuity treatments on truck corridors with nighttime pedestrian activity and near access points. Enforcement at night should focus more strongly on speed control, risky overtaking, and pedestrian conflict zones on roads with frequent heavy-vehicle movement. From an education perspective, nighttime campaigns should warn pedestrians and motorcyclists about low-visibility conflicts with heavy vehicles and encourage the use of reflective aids, safer crossing behavior, and greater caution in poorly lit freight corridors (101, 102).

## 5 Conclusion

This study examined the factors associated with injury severity in crashes involving heavy vehicles and vulnerable road users (VRUs) in Pakistan. As road traffic injuries continue to represent a major public health burden in low- and middle-income countries, identifying the factors associated with severe injury outcomes is critical for informing effective prevention strategies and strengthening evidence-based road safety policies. To address this gap, the study jointly analyzed pedestrians, motorcyclists, and motorized rickshaw users within a unified analytical framework and employed a random parameters binary probit model with heterogeneity in means and variances to capture unobserved variation in parameter effects across observations. The results show that severe injury risk is concentrated among the most exposed road users and is strongly associated with roadway and operating conditions. Pedestrians and motorcyclists were more likely to sustain severe injuries, and younger VRUs also faced a higher probability of severe injury. In addition, crashes occurring on weekends, at night, under rainy conditions, on primary roads, on two- and three-lane roads, at vertical curves, at locations with sidewalks, and near access points were associated with a higher risk of severe injury.

The results also show that the effects of nighttime and distraction are not constant across crashes. Instead, both variables varied across observations, indicating that their estimated effects vary according to the specific crash context. The effect of nighttime became stronger in pedestrian-related crashes, at access points, and under rainy conditions, while the effect of distraction became stronger when the involved VRU was a pedestrian. These findings indicate that heavy vehicle and VRU crash severity in Pakistan cannot be understood only through average effects, but must be interpreted through a context-specific public health lens that accounts for heterogeneous exposure to risk. In a mixed-traffic environment where pedestrians, motorcyclists, motorized rickshaws, and heavy vehicles often share limited road space under weak separation and inconsistent control, the same risk factor may be associated with different severity outcomes depending on the surrounding crash environment.

These results are important for two reasons. First, they show that heavy vehicle and VRU crash severity in Pakistan is associated not only with user vulnerability, but also with roadway features and environmental conditions associated with more severe injury outcomes. Second, they show that treating risk factors as fixed across all crashes can conceal important variation in their estimated effects across crash contexts, which has direct implications for public health surveillance and targeted intervention design. In this sense, the study adds to the limited evidence from low- and middle-income mixed-traffic settings and provides a more detailed understanding of the factors associated with severe injury outcomes in crashes involving heavy vehicles and different VRU groups.

This study has several limitations. First, the analysis is based on emergency-service crash records obtained from Rescue 1122 in Rawalpindi, Pakistan, which may limit the generalizability of the findings to other cities and regions. Traffic patterns, road infrastructure, vehicle composition, driver behavior, and emergency response systems in Rawalpindi may differ from those in other regions of Pakistan and other low- and middle-income countries. The findings should be interpreted within the context of the study area. Second, Rescue 1122 records include only crashes attended by the emergency service. Crashes that did not require or receive emergency assistance, particularly minor crashes, may therefore be absent from the dataset. Accordingly, the high proportion of severe injuries in the dataset, comprising 775 of the 1,007 records (76.96%), may partly reflect selection toward more serious crashes requiring emergency assistance, with minor crashes underrepresented. The completeness and accuracy of the recorded information may also vary according to the circumstances of the emergency response, which may introduce reporting bias. Injury severity was recorded at the crash scene using the information available during the emergency response. The recorded injury classification may therefore differ from a diagnosis made after a complete hospital assessment. The findings should therefore be interpreted as representing emergency-service-attended crashes rather than all crashes occurring in the study area. Third, the dataset does not include several potentially important variables, including exposure measures, operating speeds, pre-crash trajectories, visibility conditions, driver fatigue, VRU behavior, physiological characteristics, and post-crash medical response time. Although the random parameters framework captures unobserved heterogeneity, some crash-specific factors remain unavailable. Future research should examine data from multiple cities and combine emergency-service records with police and hospital data. Studies should also include more detailed behavioral, roadway, exposure, and traffic information to improve the generalizability and practical value of the findings.

## Data Availability

The data analyzed in this study is subject to the following licenses/restrictions: The data underlying this study were obtained from Rescue 1122 Rawalpindi, Punjab, Pakistan, with official permission for academic research. These data are not publicly available or deposited in a public repository because access to the raw data is controlled by Rescue 1122 and is subject to its institutional data-access and approval procedures. Researchers seeking access to the data may contact Rescue 1122 directly and submit a request in accordance with the organization’s applicable institutional procedures. Requests to access these datasets should be directed to Emergency Services Department, pio@rescue.gov.pk.
